# Fungal planktonic community related to salinity and temperature in an oligotrophic sea

**DOI:** 10.3389/fmicb.2025.1435925

**Published:** 2025-01-29

**Authors:** Ashwag A. Asseri, Alexandra Coello-Camba, Susana Agustí

**Affiliations:** Marine Science Program, The Biological and Environmental Sciences and Engineering Division, King Abdullah University of Science and Technology, Thuwal, Saudi Arabia

**Keywords:** marine fungal communities, Chytridiomycota, salinity, temperature, Red Sea, 28S

## Abstract

Marine fungi play a crucial role in carbon cycling and food webs by acting as saprophytes or parasites and shaping host communities. However, our knowledge of these fungi in the marine ecosystem remains limited. To address this gap, we conducted a study to investigate the diversity of planktonic fungal communities in the Red Sea, a warm and oligotrophic sea. We collected water samples from the photic layer at six sites along the Red Sea basin and analyzed the fungal community by targeting the 28S rRNA gene. Our results showed that Chytridiomycota dominated these communities, accounting for 85% of reads, followed by members of the divisions Basidiomycota (4.7%) and Cryptomycota (4.13%). Interestingly, we found that fungal communities did not exhibit significant changes with depth or chlorophyll concentration. However, they did vary with the latitudinal gradient in environmental conditions, which is characterized by high temperature (ranging from 22.3 to 27.0°C) and salinity (ranging from 38.0 to 40.4 PSU). Specifically, the proportions of Chytridomycetes and Neocallimastigomycetes (the two dominant classes of Chytridiomycota) were negatively correlated between themselves. Chytridomycetes exhibited a negative correlation with temperature (R^2^ = 0.60, *p* = 0.0028) and a positive correlation with salinity (R^2^ = 0.49, *p* = 0.010), being more abundant in the northern Red Sea. Conversely, Neocallimastigomycetes showed an increase in abundance with increasing temperature (R^2^ = 0.61, *p* = 0.0026) and a decrease with increasing salinity (R^2^ = 0.40, *p* = 0.026), making them more prevalent in the southern Red Sea. Overall, our study described a differential distribution of the most dominant fungal classes, with potential significance in their control of planktonic populations and consequent influence in the carbon cycle in the Red Sea ecosystem. These findings underscore the importance of further research to better understand the role of marine fungi in ecosystem functioning.

## 1 Introduction

Marine fungi are full of unique adaptive capacities that enable them to colonize a wide range of habitats, including marine and freshwater (Comeau et al., 2016; Amend et al., 2019; Grossart et al., 2019; Burgaud et al., 2022; Ilicic and Grossart, 2022; Sen et al., 2022). Fungi have migrated from marine to terrestrial settings several times, and viceversa, leading to related diversities between marine and terrestrial fungal communities (Amend et al., 2019). Numerous studies have shown that fungi and plants were the first eukaryotes to colonize the land, with mycorrhizal symbioses facilitating this (Lutzoni et al., 2018).

Marine fungi have been found in every marine habitat including sediments, mangroves, and algae (Balabanova et al., 2018; Ogaki et al., 2019). They can be found colonizing and adapting to a variety of substrates such as driftwood, mangrove wood, roots, pneumatophores, seedlings, leaves of mangrove plants (Devadatha et al., 2021), soils, and sediments in marine environments (Li et al., 2016; Marchese et al., 2021), seawater and water column (Wang et al., 2018), invertebrates (Yarden, 2014; Calabon et al., 2019), other animals (Hyde et al., 1998; Danesi et al., 2021) and dead and decomposing animal substrates (Kohlmeyer and Kohlmeyer, 1979; Hyde et al., 2000). Several species have been recorded from marine habitats such as coastal oligotrophic and upwelling waters, deep-sea sediments, and anoxic zone sediments (Damare and Raghukumar, 2008; Gutiérrez et al., 2016; Lear et al., 2018). Despite fungi being essential components of the marine environment, they are understudied compared to other microorganisms. The fungal communities’ abundance and ecological function in the marine environment in many regions remain little explored.

Three phyla, Ascomycota, Basidiomycota, and Chytridiomycota are predominant and globally represented in the marine fungi (Hassett et al., 2020a, 2020b). The majority of marine fungi can be represented by the large ribosomal subunit (LSU) 28S rRNA gene, followed by the internal transcribed spacer (ITS) region (Hassett et al., 2019). The Phylum Chytridiomycota (chytrids), are at the base branch of the Kingdom Fungi and are the most common parasites in plankton communities, representing the dominant parasites in both terrestrial and aquatic ecosystems (Sime-Ngando, 2012). Chytrids, however, are classified as obligate parasites, obligate saprophytes, or facultative parasites. These fungi can severely depress their host populations and infect a wide variety of hosts, including fish, zooplankton, and eggs, but primarily phytoplankton and are often found to infect diatoms (Gleason et al., 2008; Grami et al., 2011; Grossart et al., 2019; Frenken et al., 2020; Fisher et al., 2021). Nevertheless, it is unclear whether the degree of parasitism or saprophytism is related to individual taxa or whether chytrids exhibit a range of strategies, ranging from obligate parasitism to obligate saprophytism or facultative parasitism lifestyles, depending on environmental conditions (Frenken et al., 2017).

It has been postulated that salinity may influence the distribution of planktonic fungi communities (Sime-Ngando, 2012; Hassett and Gradinger, 2016). In a recent global ocean study, environments with atypical salinity regimes hosted higher proportions of Chytridiomycota, relative to open oceans (Hassett et al., 2020a, 2020b). The Red Sea is one of the most saline water bodies in the ocean (Edwards, 1987), with typical concentrations ranging from 36 to 40.5 PSU along a south-to-north gradient (Sofianos and Johns, 2015). It is also one of the warmest seas on Earth with surface seawater temperature ranging from 22°C to 32°C, characterized by a gradient of decreasing temperature from south to north (Rasul et al., 2015). This semi-enclosed basin displays overall oligotrophic conditions, although nutrient concentrations increase toward the south, where Indian Ocean nutrient-enriched intermediate waters enter the basin through the Gulf of Aden (Churchill et al., 2014). This latitudinal pattern is reflected in increasing primary production in the southern Red Sea (López-Sandoval et al., 2021). The Red Sea lacks inputs from rivers or stream sources, and nutrients become available either through vertical mixing, aerial deposition, and intrusion of water masses (Raitsos et al., 2013). The general water column of the Red Sea is highly stratified, with thermocline located from 50 to 250–300 m in depth. Previous studies on fungi in the Red Sea are scarce and have mainly focused on the diversity and identification of fungi in mangroves and seaweeds (Abdel-Wahab, 2005; Abdel-Gawad et al., 2014; Abdel-Wahab et al., 2019). According to Simões et al. (2015), studies on Red Sea mangroves revealed the presence of Ascomycota, Basidiomycota, and mitosporic fungi. The presence of terrestrial fungi such as *Aspergillus* sp. and *Penicillium* sp., as well as *Fusarium* sp*., Neurospora* sp., and *Rhizopus* sp., was discovered in studies of fungal communities in the coastal Red Sea (Basem et al., 2012; Abdel-Wahab et al., 2014; Alwakeel, 2017). Other coastal studies identified *Candida* spp., *Cryptococcus* spp., *Debaryomyces* spp., and *Rhodotorula* spp. in seawater (Abd-Elaah, 1998). Eight fungal genera (*Aspergillus, Penicillium, Thielavia, Fusarium, Emericella, Cladosporium, Scytalidium and Alternaria*) belonging to the division Ascomycota were identified in samples from the nearshore of the city of Jeddah (Saudi Arabia, Alwakeel, 2017). Based on metagenomic approaches, Hassett et al. (2020a, 2020b) revealed that Chytridiomycota dominated fungal communities in the surface waters of the Red Sea.

Despite the key role parasitic and saprophytic fungi play in pelagic ecosystems (Sime-Ngando, 2012; Frenken et al., 2020; Klawonn et al., 2021), few of the previous studies have explored the fungal communities associated with plankton in the warm, oligotrophic and salty Red Sea waters. In the present work, we aim to characterize, for the first time, the diversity and geographical distribution of planktonic fungal communities in the oligotrophic waters of the Red Sea. We analyzed plankton samples in six stations sampled along a latitudinal gradient to encompass the variability in oceanographic conditions of the Red Sea. CTD cast profiles were conducted at each station, sampling the water column from the surface to the base of the photic layer to characterize environmental variability. Planktonic fungal 28S analyses focused on two specific depths, the surface and the deep chlorophyll maximum (DCM), due to the distinct water column properties at these layers (e.g., light, temperature, and nutrient availability), which drive significant changes in phytoplankton abundance, production, community structure, as well as microbial and carbon processes in the Red Sea (Qurban et al., 2017; Al-Otaibi et al., 2020). While surface and DCM layers have been shown to shape the taxonomic diversity of the pelagic mycobiome in other ecosystems (Hassett et al., 2019), their influence on the distribution of the pelagic mycobiome in the Red Sea remains unexplored. We applied next-generation sequencing targeting 28S rRNA genes region, and identified the presence of these parasites along the basin, investigated the community composition distribution in relation to environmental variability, and evaluate their potential effect on host populations. We also aim to assess the dominance of the Chytridiomycota phylum within the community, as it is anticipated to be influenced by salinity gradients.

## 2 Materials and methods

### 2.1 Study site and sampling

**Figure 1:**
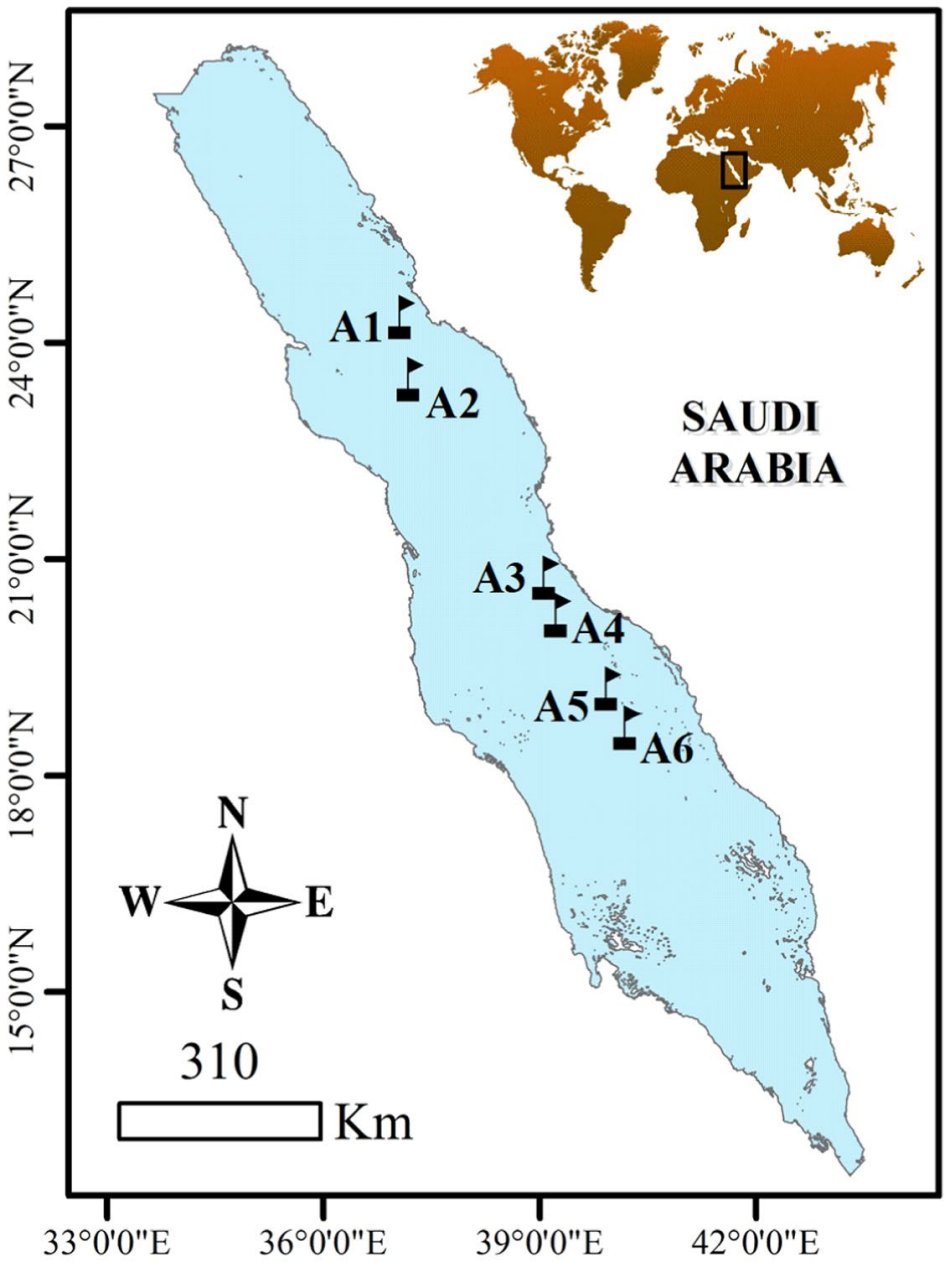
Locations of sampling stations in the Red Sea visited from the 3rd to the 8th of April 2019.

The sampling was conducted between the 3rd and the 8th of April 2019 during a cruise (Deep Cruise) on board R/V Thuwal in the north, south, and central Red Sea (Figure 1). A total of six stations were selected from 18.67 to 24.46 °N to include the latitudinal gradient in oceanographic conditions characterizing the Red Sea. Vertical profiles were conducted using a Sea-Bird© Electronics 911 plus CTD that was equipped with an oxygen sensor, fluorometer, turbidity meter, and PAR sensor (Biospherical/LI-COR, SN 1060) at all sampled stations. Water samples were collected at 7–8 different depths, ranging from the surface to the bottom of the photic layer (150 meters) using 12 L Go-Flo Niskin bottles that were attached to the CTD-rosette system. Seawater temperature and salinity data were obtained from the CTD casts, and the deep chlorophyll maximum (DCM) was identified by the fluorescence peak (Cai et al., 2022).

Water samples were analyzed for nutrients, chlorophyll *a* (Chl-*a*) concentration, and sequencing for fungi communities. Chl-*a* concentration was measured by filtering 0.5 L of water onto 2.5 cm Whatman^®^ GF/F filters at the various depths sampled. The filters were then submersed in 90% acetone in the dark for 24 h and Chl-*a* concentration was measured in a Trilogy^®^ fluorometer (Turner Designs, CA) following the methods described in López-Sandoval et al. (2021). Finally, nutrient analyses were conducted using a SEAL AA3 Segmented Flow Analyzer (SEAL Analytical), and standard autoanalyzer methods (Hansen and Koroleff, 1999) were employed for the analysis.

### 2.2 Phylogenetic analysis of Red Sea planktonic fungi

#### 2.2.1 Sampling process

Seawater samples were collected from the rosette Niskin bottles at a depth of 5 m and at the depth of the DCM. The collected samples were transferred to an acid-cleaned carboy and between 8 and 10 liters were immediately filtered through replicated Isopore membrane filters with a pore size of 3 μm and a diameter of 47 mm using a Masterflex peristaltic pump (Millipore Corporation). The filtered samples were then placed in Falcon tubes (15 mL) and stored at −80°C until analysis on land. To avoid any contamination between samples, the filtration systems were cleaned using bleach (10%) and filtered seawater.

#### 2.2.2 DNA extraction

The DNA extraction process for all samples involved bead-beating methods and followed the manufacturer’s instructions for the DNeasy^®^ PowerWater^®^ DNA Extraction kit. The DNA quantities obtained after extraction were measured using a Qubit^®^ fluorometer (Life Technologies, Carlsbad, CA, USA) before proceeding with gene-specific PCR amplification.

#### 2.2.3 PCR amplification

The 28S rRNA gene sequence was targeted using LR0R (5’-ACCCGCTGAACTTAAGC-3′) andLR2 (5’-ACTTCAAGCGTTTCCCTTT-3′) primers (Hassett et al., 2017), which were purchased from Sigma-Aldrich^®^ with Illumina overhang adaptors attached (Forward: 5’-TCGTCGGCAGCGTCAGATGTGTATAAGAGACAG-*specific locus*; Reverse: 5’-GTCTCGTGGGCTCGGAGATGTGTATAAGAGACAG-*specific locus*). The PCR protocol included an initial activation step at 95°C for 15 min, followed by 30 cycles of 95°C melting for 1 min, 50°C annealing for 30 s, and 72°C extension for 90 s, with a final extension step at 72°C for 5 min, as described in Hassett et al. (2017). We used the Qiagen multiplex PCR master mix (QIAGEN, Valencia, CA, USA) for PCR amplification. The PCR products were checked for amplification of the targeted sequences by gel electrophoresis (2 g of agarose in 100 mL of TAE). Amplicons were then cleaned using AMPure XP magnetic beads (Beckman Coulter, Brea, CA, USA).

#### 2.2.4 Library preparation

After PCR amplification and cleaning, a sequencing library was prepared by adding NextEra^®^ XT Indexes using a second PCR following the Illumina protocol. The resulting amplicons were then subjected to another round of cleaning using AMPure magnetic beads, and quantified using a Qubit fluorimeter. The samples were then pooled at KAUST CORELab facilities according to the Illumina MiSeq protocol and quantified using qPCR. The pool sizes were verified using a Bioanalyzer from Agilent Technologies. The resulting pool, containing samples with a concentration of 14.17 μM, was sequenced on Illumina MiSeq using 2×300 bp paired-end reads with MiSeq reagent kit v3 (Illumina, Inc.), with 25% PhiX.

#### 2.2.5 Taxonomic annotation of the 28S ASVs

We processed the sequencing data by first removing the primers from the forward and reverse sequences using the *cutadapt* tool (Martin, 2011). The resulting trimmed sequences were then quality-filtered, dereplicated, merged, and sorted into Amplicon Sequence Variants (ASVs) following the DADA2 pipeline (Callahan et al., 2016) within RStudio.^1^ To assign taxonomic classifications to the ASVs, we constructed a reference database containing 164 28S fungal sequences that had been previously classified to the species level whenever possible, based on molecular taxonomic studies (Karpov et al., 2010; Richards et al., 2012; Seto et al., 2017; Hassett et al., 2017; Jones et al., 2019). These sequences were obtained from the European Nucleotide Archive (ENA).^2^

### 2.3 Statistical analysis

Statistical analyses were conducted using JMP Pro (v. 15.0) and Origin Pro^®^ 2021 (v.9.8). Mean and standard error calculations were performed. To test for differences between depths, we applied ANOVA with the *post-hoc* Tukey HSD Test. The relationships between the fungi community and environmental parameters were analyzed using Spearman correlation and linear regression. Statistical significance was set at *p* < 0.05.

## 3 Results

### 3.1 Environmental parameters

**Figure 2:**
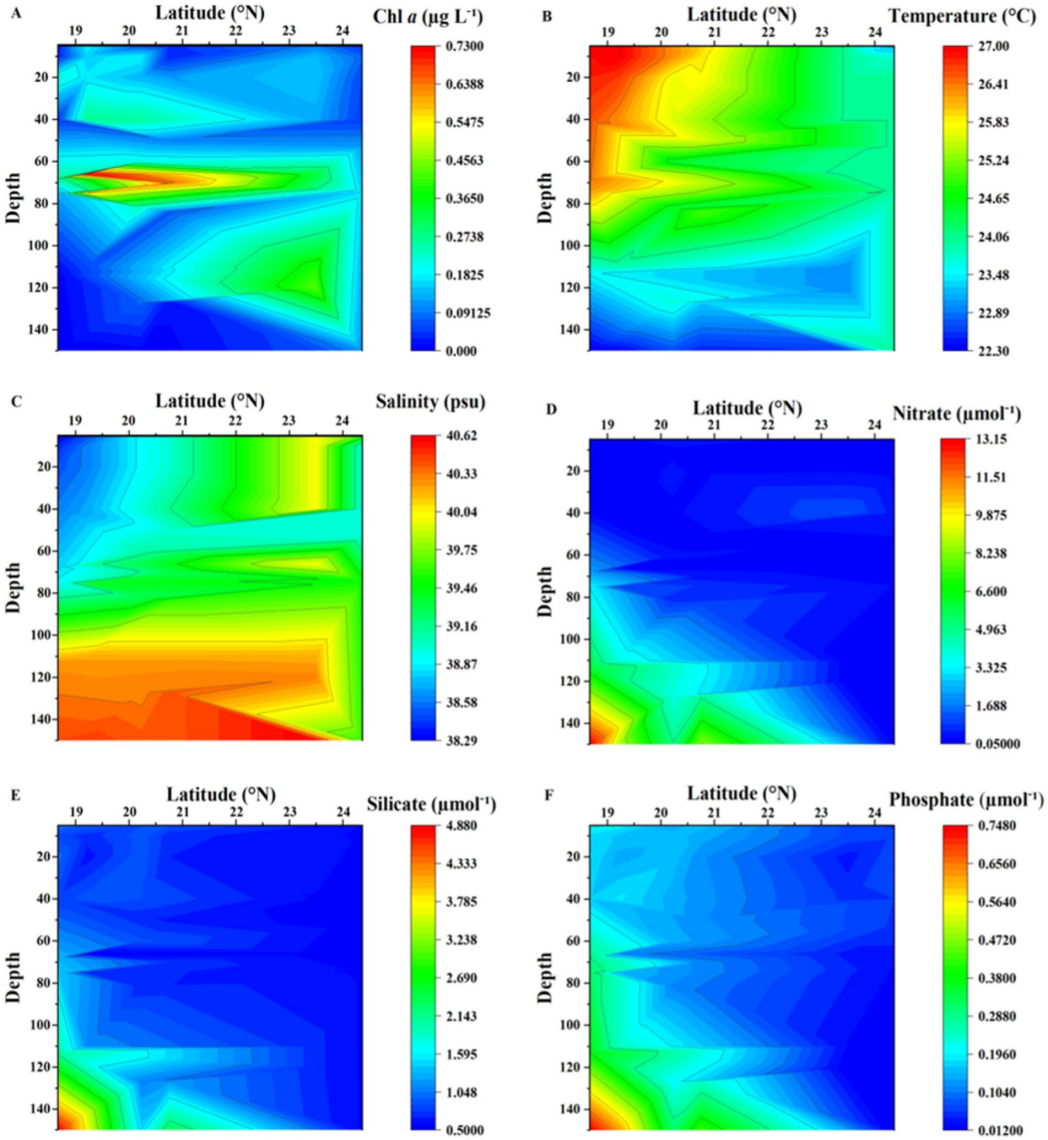
Contour plots showing the distribution of **(A)** chlorophyll-*a* (Chl-*a*), **(B)** Temperature (°C), **(C)** Salinity (PSU), **(D)** Nitrate (μmol^−1^), **(E)** Silicate (μmol^−1^), and **(F)** Phosphate (μmol^−1^) from sampling stations in the Red Sea.

Chlorophyll-*a* concentrations (Chl-*a*) were low, displaying low variability. The lowest value of 0.057 μg L^−1^ was recorded at the surface in the northernmost station with concentrations increasing toward the southern waters (Figure 2A). The highest Chl-*a* values were observed at the deep chlorophyll maximum (DCM), located between 60 and 80 m depth, ranging from 0.54 to 0.73 μg L^−1^ (Figure 2A). Surface water temperatures ranged from 22.3°C to 27.0°C, with a clear southward increase, while the salinity varied from 38.0 to 40.4 PSU, showing higher values in the northern stations with a strong vertical gradient toward the southern stations; both temperature and salinity profile suggest some mixing at the northernmost station where strong winds were found at the time of sampling (Figures 2B,C). Nutrient concentrations from the surface to the 150 m depth examined ranged from 0.05 to 13.1 μmol^−1^, 0.55 to 4.8 μmol^−1^, and 0.013 to 0.74 μmol^−1^, for nitrate, silicate, and phosphate, respectively. Nutrient concentrations increased with depth, with shallower nutrient-rich layers observed at the southern stations (Figures 2D–F). The south to north gradient in nutrients concentration at the surface waters was more gradual for phosphate (Figure 2F).

### 3.2 Fungal community composition

**Figure 3:**
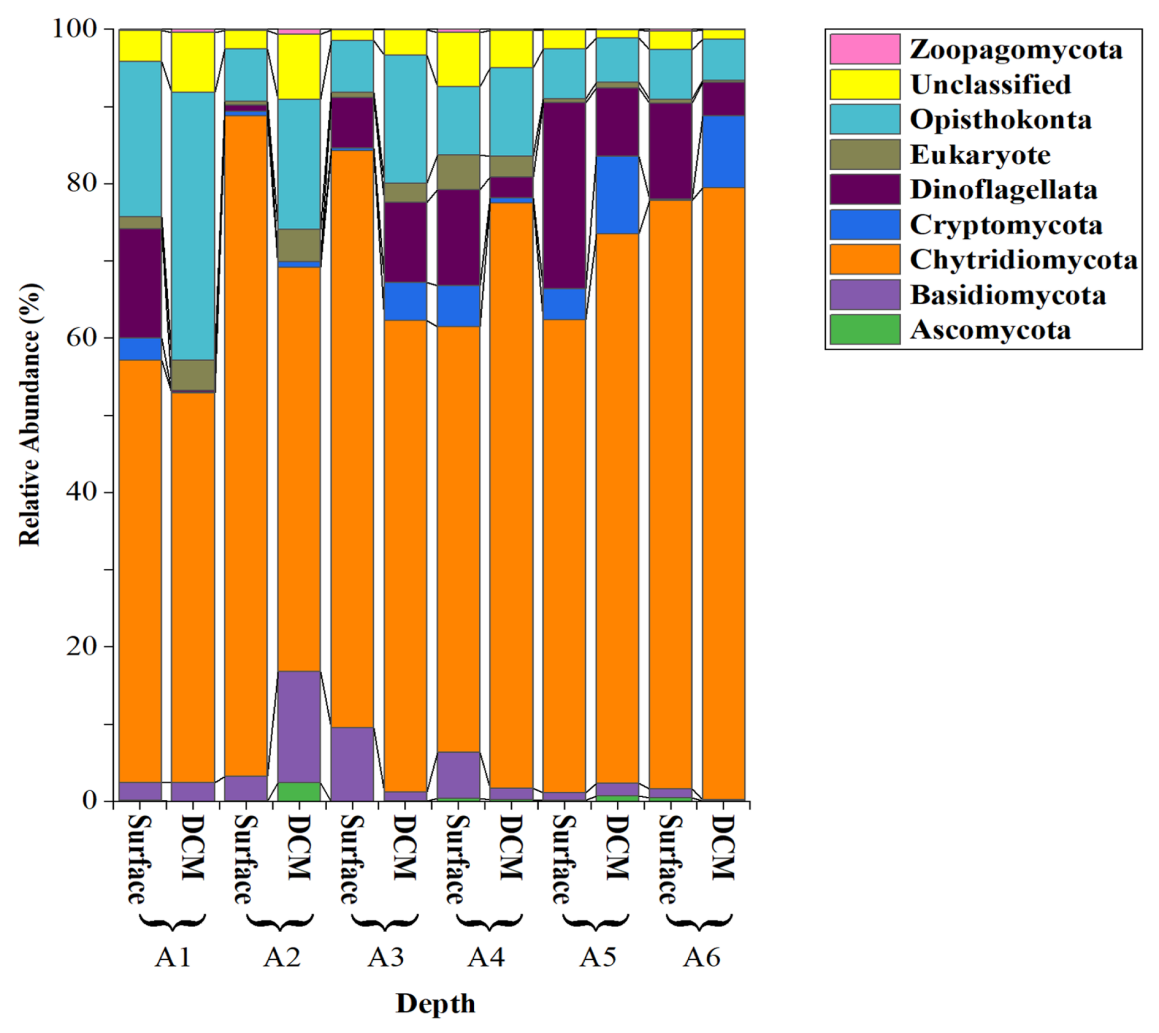
Relative abundance of the fungi Phyla and other close Phyla found in the epipelagic layer of the Red Sea based on the analysis of the 28S rRNA amplicon.

28S sequencing and processing of our 12 samples resulted in 10,409,742 raw amplicons, leading to 3,480 fungal amplicon sequence variants (ASVs), after the analysis with DADA2 (Supplementary Figure S1). Our analysis of the Red Sea fungal community revealed a diverse group of 98 identified species, distributed across 11 classes, 19 orders, and 48 families (Supplementary Figure S1). However, we also identified 1,417 unclassified taxa, which we addressed using the NCBI BLAST tool to focus on the most abundant groups. This approach enabled us to identify 73 taxa belonging to five groups (Ascomycota, Basidiomycota, Chytridiomycota, Cryptomycota or “Rozellomycota,” and Zoopagomycota) (Figure 3).

**Figure 4:**
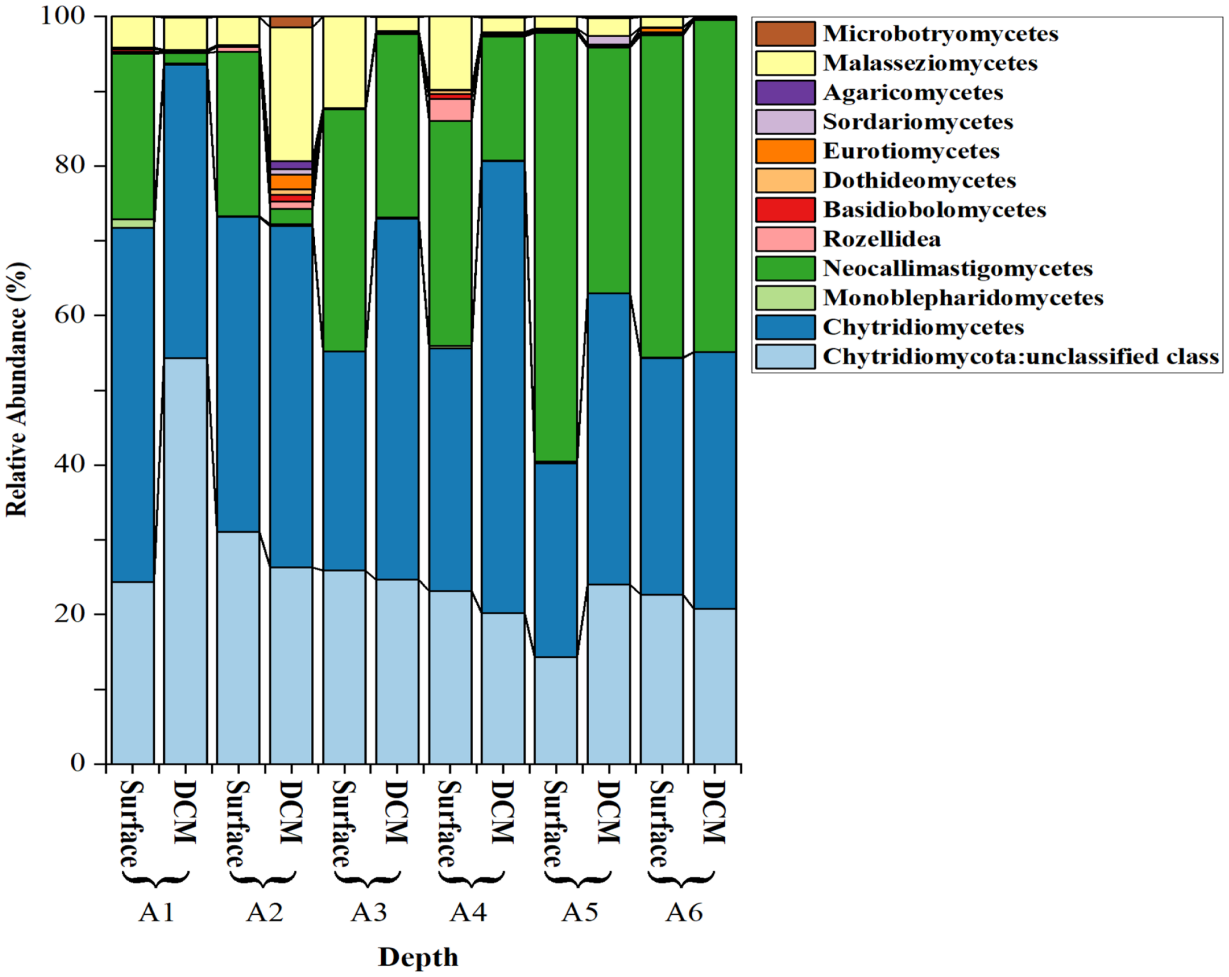
Relative abundance (%) of selected fungal classes across different phyla and depths in the stations sampled, including three classes in Chytridiomycota, one in Zoopagomycota and Cryptomycota, and three in Basidiomycota and Ascomycota.

The most abundant fungal isolates were from the division Chytridiomycota, representing 85.16 ± 2.30% (mean ± SE) of the total sequences, followed by Basidiomycota (4.70 ± 1.50%) and Cryptomycota (4.13 ± 1.22%). Zoopagomycota and Ascomycota were less abundant (< 2%; Figure 3). Among the Chytridiomycota, the most abundant classes were Chytridomycetes (39.63 ± 2.80%), Neocallimastigomycetes (27.00 ± 3.20%), and Monoblepharidomycetes (0.21 ± 0.05%) (Figure 4). The most abundant class within Basidiomycota was Malasseziomycetes (5.15 ± 1.50%) (Figure 4). The genus Rozella (Class Rozelidea, Figure 4) represented the only identified taxon within Cryptomycota. Ascomycota was less abundant and was dominated by the classes Eurotiomycetes, Sordariomycetes, and Dothideomycetes (Figure 4).

**Figure 5:**
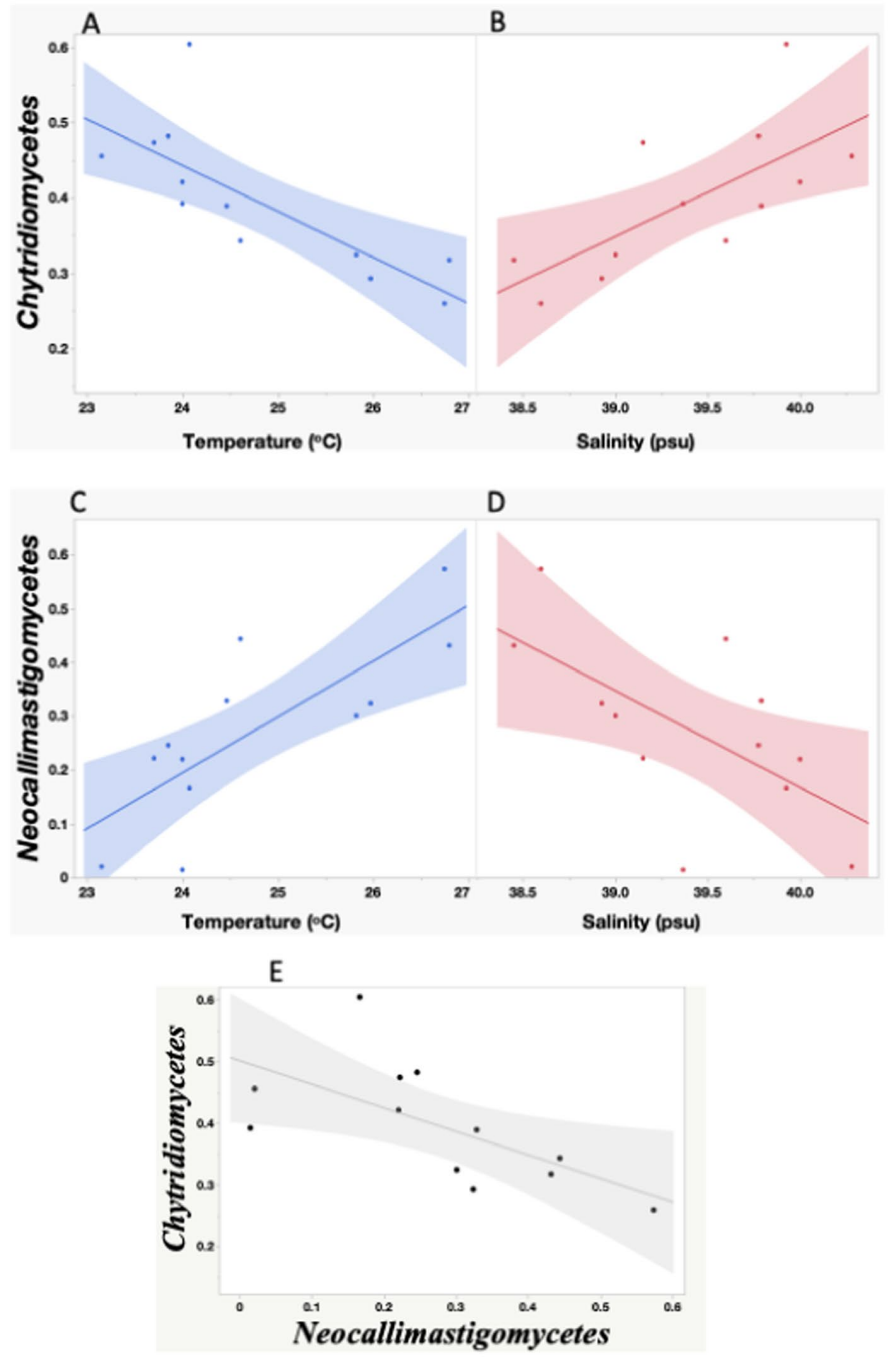
Linear relationships between seawater temperature and salinity and the proportion of Chytridiomycetes **(A,C)**, and Neocallimastigomycetes **(B,D)**, and between Neocallimastigomycetes and Chytridiomycetes proportion observed during the study. The red, blue and black lines represent the linear regression fits for: **(A)** Chytridiomycetes = 1.91–0.06* Temperature °C; **(C)** Neocallimastigomycetes = −2.296 + 0.103*Temperature °C; **(B)** Chytridiomycetes = −4.236 + 0.117*Salinity; **(D)** Neocallimastigomycetes = 7.342–0.179*Salinity; E- Chytridiomycetes = −1.09 + 0.706*Neocallimastigomycetes. All the relationships are significant (**A,B,E**: *p* < 0.05; **C,D**: *p* < 0.005). The shadow areas encompass the 95% error intervals of the different regressions.

Chytridiomycetes and Neocallimastigomycetes were negatively correlated (Spearman’s *ρ* = −0.72, *p* = 0.0082). Neocallimastigomycetes was negatively correlated with latitude (ρ = −0.87, *p* = 0.0002) and with the proportion of the Basidiomycota class Malasseziomycetes (ρ = −0.61, *p* = 0.033), indicating an increase in the southern Red Sea (Figure 5). In contrast, Chytridiomycetes showed a higher proportion in the northern stations but were not significantly related to latitude.

We also investigated the relationship between fungal classes and environmental variables. Chytridiomycetes showed a negative relationship with temperature (R^2^ = 0.60, *p* = 0.0028) and a positive relationship with salinity (R^2^ = 0.49, *p* = 0.010), while Neocallimastigomycetes showed a positive relationship with temperature (R^2^ = 0.61, *p* = 0.0026) and a negative relationship with salinity (R^2^ = 0.40, *p* = 0.026). The study did not find significant differences in the fungal community composition with depth, whether considering Phyla or Classes. Additionally, the abundance of fungi was not directly linked to changes in primary productivity, as evidenced by the lack of a relationship between the fungal community and Chl-*a* concentration.

## 4 Discussion

The fungal community in terrestrial environments has been extensively researched and documented (Bass and Richards, 2011; Zanne et al., 2020). However, studies on the diversity and ecological role of fungi in aquatic environments have been comparatively scarce but have progressed in recent years (Lai et al., 2007; Jones et al., 2019; Grossart et al., 2019; Klawonn et al., 2021). In this study, we aimed to investigate the distribution of the planktonic marine fungal community in the oligotrophic, saline, and warm waters of the Red Sea (Saudi Arabia). To achieve this, we utilized 28S sequencing to analyze fungal communities at different stations along the Red Sea. Our findings shed new light on the understudied marine fungal community in the region and contribute to a better understanding of the microbial diversity and ecology of marine environments (Gittings et al., 2019).

Our results agree with previous studies reporting the distribution of chlorophyll-*a* and nutrient concentrations in the Red Sea (Coello-Camba and Agustí, 2021). Chlorophyll-*a* concentrations ranged between 0.54 and 0.73 μg L^−1^ at the depth of the DCM, which is often considered the boundary between the surface and deep ocean. The concentrations of chlorophyll-*a* increased at the DCM, indicating a significant amount of primary productivity occurring in this zone. The higher values of chlorophyll-a in the south may be attributed to factors such as temperature, light availability, and nutrient availability (Gittings et al., 2019). The concentrations of essential nutrients such as nitrate, silicate, and phosphate also varied at different depths and locations in the Red Sea, ranging from 0.25–13.1 μmol^−1^, 0.55–4.8 μmol^−1^, and 0.013–0.74 μmol^−1^ for nitrate, silicate, and phosphate, respectively, and were found to be higher in the southern Red Sea stations sampled (Gittings et al., 2019). All these variables highlight the importance of studying physical and chemical factors that influence primary productivity in marine ecosystems. Understanding the distribution of nutrients and chlorophyll-*a* concentrations at different depths and locations can provide insights into the dynamics of marine ecosystems and help inform management and conservation efforts. As an oligotrophic sea, picocyanobacteria dominates the phytoplankton community but nutrients inputs favors the growth of nano and microphytoplankton (i.e., diatoms) that tend to be more abundant in the Southern Red Sea (Qurban et al., 2017). However, our understanding of the Red Sea remains limited with few reports documenting the seasonality and shifts in plankton communities in the open waters. Raitsos et al. (2013) based on decade-long satellite data, identified seasonal patterns in surface Chl-*a*, with maximum concentrations during winter and minimal in summer. However, they found that Chl-a concentration does not increase regularly from north to south, as the pattern is perturbed by the formation of wind induced eddies along the Red Sea basin (Raitsos et al., 2013; Kürten et al., 2016). Mesoscale eddies have been shown to influence the patterns in primary production and plankton communities’ composition along the Red Sea basin (Kürten et al., 2016; Qurban et al., 2017).

Several authors have pointed out that the 28S has a higher variability compared to the 18S rRNA gene sequence, making it more useful for the taxonomic resolution of fungi (Schoch et al., 2012; Hassett et al., 2020a, 2020b). While eukaryotic microbial community analysis is primarily based on polymerase chain reaction (PCR) amplification of the 18S, it can also amplify numerous stretches of the bacterial 16S gene, which can hinder the high-throughput detection of rare eukaryotic species (Machida and Knowlton, 2012; Kounosu et al., 2019). The 28S sequence is the structural ribosomal RNA for the large subunit (LSU) of eukaryotic cytoplasmic ribosomes, and thus, it is one of the basic components of all eukaryotic cells. Moreover, LSU is the favored phylogenetic marker for mycologists (Schoch et al., 2012) due to its ability to resolve higher taxonomic ranks and its relatively conserved regions that can be used for designing PCR primers. The use of 28S sequencing in this study, therefore, offers greater resolution and accuracy for the identification and characterization of fungal communities in the marine environment. In our 28S-based survey of fungal communities, we found a proportion of sequences corresponding to choanoflagellates (Opisthokonte) among other unclassified sequences, which is not unexpected, given that fungi are evolutionarily related to choanoflagellates and animals, as they share a common ancestor. Among the identified fungal phyla, Chytridiomycota (chytrids) were the most abundant in our study. Chytrids are known to be predominant in aquatic environments, and their cell structure has much in common with that of protists (Sime-Ngando, 2012). Previous studies have also reported the dominance of Chytridiomycota in marine ecosystems as detected by high-throughput sequencing surveys (Picard, 2017; Hassett et al., 2020a, 2020b). However, other studies have reported the dominance of Ascomycota and Basidiomycota in marine plankton (Morales et al., 2019), but these findings may have been constrained by the focus on specific genes. The choice of genetic marker region and sequence databases used can also impact the results (Frenken et al., 2020), highlighting the importance of careful consideration when designing and interpreting molecular surveys of microbial communities. Overall, the results of our study add to the growing body of evidence regarding the diversity and ecological importance of fungal communities in marine environments. In our study, we found that the Chytridiomycota classes Chytridomycetes and Neocallimastigomycetes were the most abundant among the identified chytrids. Chytridomycetes are common in aquatic environments (Sime-Ngando, 2012; Frenken et al., 2017), and are ecologically important as parasites of phytoplankton, infecting various species of phytoplankton, especially diatoms, regulating community composition, transferring energy via the mycoloop, and supporting nutrient cycling (Kagami et al., 2014; Gleason et al., 2017; Grossart et al., 2019). Neocallimastigomycetes are anaerobic fungi typically found in the digestive tracts of herbivorous mammals where they play an important role in the degradation of plant material (Hess et al., 2020). They have being found in the marine environment in the guts of some herbivores (i.e., sea urchin and iguana; Gleason et al., 2017) and in coastal plankton and sediments samples (Picard, 2017). However, infer the ecological roles of these understudied fungi in the pelagic ocean is still challenging. Interestingly, we also identified Cryptomycota, represented by the genus Rozella spp., which is a relatively newly described group of fungi. Cryptomycota are characterized by the absence of chitinous cell walls, which is a unique feature among fungi. They have been found in a variety of environments, including freshwater, marine, and soil habitats, and have been shown to play important roles in nutrient cycling (Jones, 2011; Gleason et al., 2017). The identification of Cryptomycota in our study highlights the potential importance of this understudied group of fungi in marine ecosystems.

Recent global studies have revealed significant variability in fungal diversity within oceanic regions, which can be attributed to changes in physicochemical parameters such as temperature and salinity. Studies conducted by Shearer et al. (2007), Tisthammer et al. (2016), Morales et al. (2019), and Hassett et al. (2020a, 2020b) have all reported on this phenomenon. Tisthammer et al. (2016) demonstrated that a range of environmental variables, including temperature, salinity, dissolved oxygen, nitrate, phosphate, silicate, and depth, played a significant role in shaping marine fungal communities. Their analyses revealed that environmental variables accounted for 73% of the total composition variance, compared to only 18% attributed to geographic location. These findings highlight the importance of physicochemical parameters in shaping marine fungal communities and the need for further research to better understand their ecological implications.

During the global study performed by Hassett et al. (2020a, 2020b), environments with atypical salinity regimes (<5 standard deviations from the global mean) such as the Red Sea, Baltic Sea, and sea ice, were found to host higher proportions of the Chytridiomycota relative to open oceans. The role of salinity in conditioning marine fungal distributions has been observed before; Rojas-Jimenez et al. (2019) found contrasting distributions of several Chytridiomycota orders along a salinity gradient in the Baltic Sea. Our study confirmed that Chytridiomycota is the dominant class in the photic layer of the Red Sea. Furthermore, we have found that the two dominant classes within Chytridiomycota, Chytridiomycetes and Neocallimastigomycetes, exhibit contrasting distribution patterns in relation to changes in salinity and temperature. Interestingly, we observed that Chytridiomycetes were positively correlated with salinity, suggesting that they thrive in environments with higher salt concentrations. On the other hand, we found a negative correlation between Chytridiomycetes and temperature, indicating that these fungi may prefer cooler water temperatures.

In contrast, Neocallimastigomycetes showed an opposite trend. We found an increase in their proportion toward the northwestern region of the Red Sea, together with a positive correlation between temperature and the abundance of Neocallimastigomycetes, suggesting that these fungi prefer warmer waters. Furthermore, we found a negative correlation between the abundance of Neocallimastigomycetes and salinity, indicating that they may be less prevalent in high-salinity environments.

Previous studies have shown that some chytrids have high tolerance to high temperatures, with maximum growth varying from 30 to 40°C (Gleason et al., 2008). Despite the observed patterns in relation to environmental parameters, seasonal or oceanographic disturbances can also shape these fungal communities (Townsend et al., 2003; Hassett et al., 2020a, 2020b). Additionally, host distributions and biological interactions can also influence fungal community structure (Menge and Sutherland, 1976; Hassett et al., 2020a, 2020b), as well as seasonal changes (Marquardt et al., 2016; Hassett et al., 2020a, 2020b). While nutrient inputs in the Southern Red Sea promote primary production and diatoms growth, a major potential hosts, there are wind induced processes (i.e., mesoscale eddies and vertical mixing, Raitsos et al., 2013), influencing productivity and plankton community composition, making it difficult to infer the distribution of planktonic communities in this study from environmental parameters measured alone.

Overall, these findings highlight the complex interactions that shape fungal communities in the Red Sea, with a range of environmental and biological factors playing important roles. Further research is needed to better understand the underlying mechanisms and ecological implications of these interactions.

## 5 Conclusion

This study aimed to investigate the distribution of the planktonic marine fungal community in the Red Sea and to contribute to a better understanding of the microbial diversity and ecology of marine environments. The use of 28S sequencing in this study offers greater resolution and accuracy for the identification and characterization of fungal communities in the marine environment. We found that Chytridiomycota were the most abundant phylum of fungi in the Red Sea marine environment, with Chytridomycetes and Neocallimastigomycetes being the most abundant classes among the identified chytrids. Overall, the results of this study add to the growing body of evidence regarding the diversity and ecological importance of fungal communities in marine environments.

## Data Availability

The datasets presented in this study can be found in online repositories. The names of the repository/repositories and accession number(s) can be found at: http://purl.org/phylo/treebase/phylows/study/TB2:S30578, TB2:S30578.

## Footnotes

^1^ www.rstudio.com

^2^ https://www.ebi.ac.uk/ena/browser/home
