# Supplementary material for: Fungal planktonic community related to salinity and temperature in an oligotrophic sea
Source: Front Microbiol. 2025 Jan 29;16:1435925. doi: 10.3389/fmicb.2025.1435925 (PMC11814442; doi:10.3389/fmicb.2025.1435925)
Supplement: Supplementary file 1 [file Data_Sheet_1.docx]

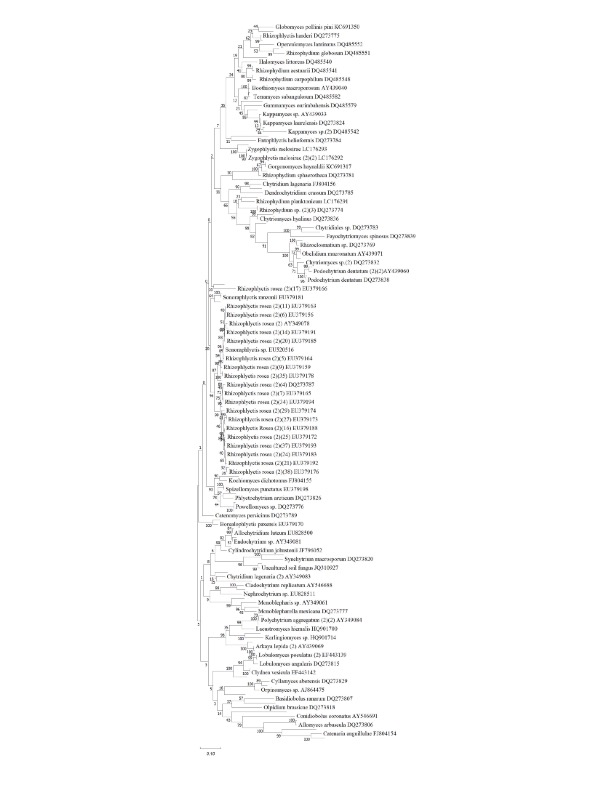


**Supplementary Figure S1.**Phylogenetic tree analysis of 164 marine fungal strains compiled in our database, based on DNA-28S sequence analysis constructed by maximum likelihood method. Scale bar shown 10% estimated sequence divergence.
